# Multi-layer adaptation of group coordination in musical ensembles

**DOI:** 10.1038/s41598-019-42395-4

**Published:** 2019-04-10

**Authors:** Pauline M. Hilt, Leonardo Badino, Alessandro D’Ausilio, Gualtiero Volpe, Serâ Tokay, Luciano Fadiga, Antonio Camurri

**Affiliations:** 1 IIT@UniFe Center for Translational Neurophysiology of Speech and Communication, Istituto Italiano di Tecnologia, Ferrara, Italy; 2 Section of Human Physiology, Università di Ferrara, Ferrara, 44121 Italy; 3 Casa Paganini—InfoMus, DIBRIS, Universita degli Studi di Genova, Viale Causa 13, Genova, 16145 Italy; 4 Philarmonie de Chambre Tokay, Paris, France

**Keywords:** Social behaviour, Human behaviour

## Abstract

Group coordination passes through an efficient integration of multimodal sources of information. This study examines complex non-verbal communication by recording movement kinematics from conductors and two sections of violinists of an orchestra adapting to a perturbation affecting their normal pattern of sensorimotor communication (rotation of half a turn of the first violinists’ section). We show that different coordination signals are channeled through ancillary (head kinematics) and instrumental movements (bow kinematics). Each one of them affect coordination either at the inter-group or intra-group levels, therefore tapping into different modes of cooperation: complementary versus imitative coordination. Our study suggests that the co-regulation of group behavior is based on the exchange of information across several layers, each one of them tuned to carry specific coordinative signals. Multi-layer sensorimotor communication may be the key musicians and, more generally humans, use to flexibly communicate between each other in interactive sensorimotor tasks.

## Introduction

Successful human-to-human interaction requires important behavioral adaptation, as well as prediction. A large body of literature has focused on cooperation towards shared goals, where humans must combine available sensory information with internal movement production models^1–4^. In this regard, researchers investigated how dyads achieve interpersonal simple sensorimotor coordination, such as walking side-by-side^5^ or rocking in rocking-chairs^6^. In such contexts, co-actors continuously influence each other and tend to spatially and temporally synchronize their movements. Beside imitation, action complementarity play a key role in inter-individual coordination with the goal of achieving efficient collaboration^7^. Social interaction indeed goes beyond synchronization with other’s actions and relies also on inferring others’ motor goals and intentions to generate a context-appropriate action. To achieve fast inter-individual coordination, individuals may build internal predictive models of other’s behavior. In function of the context, the most appropriate motor model is compared with the current observed movement, to generate a prediction error^4^ and update own motor planning^8^.

Due to the technical and analytical complexity in exploring the details of human sensorimotor interaction, only few experiments went further than a dyadic set-up^9–12^. However, in daily life, things are usually much more complex. For instance, during a conversation, information is sampled through multiple channels (e.g. vision, audition), sometimes in parallel (e.g. information in the foreground and information from the background) and at different temporo-spatial scales (e.g. slow whole-body movements versus fast lip motions). At the same time, different kinds of information may be conveyed in parallel through different channels. For example, in speech, bodily gestures and spoken words are generally co-expressive^13^. In this context, communication requires flexible means to integrate multimodal data, across multiple timescales and act accordingly. Therefore, proper quantification of (realistic) group coordination is today one of the key missing elements to understand how humans manage to interact with others by efficiently selecting, processing and sending information.

In this context, ensemble musicians have been proposed as an ideal model, by keeping the key multidimensional properties of natural sensorimotor interaction, but allowing relatively good experimental control^14,15^. Few previous studies, by relying on kinematic recordings, have started to model sensorimotor information flows across musicians. D’Ausilio and collaborators^16^ recorded violinists’ and conductors’ movement kinematics to investigate causal relationships across musicians. They showed that conductors influenced communication between musicians and that aesthetic appreciation was dependent on the co-regulation of leader-to-musician and musician-to-musician communication patterns^16^. Leadership in the orchestra scenario is explicit since the conductor determines tempo, selects musicians, leads rehearsals, and takes critical decision about interpretation of the pieces. For instance, visual cues derived from conductors’ gestures improve temporal anticipation and synchronization performance for individuals with or without musical training^17^. In the absence of explicit leadership (e.g. quartet), this role is shared across musicians^18^. The quartet scenario was also used by Chang and collaborators^19^ to investigate the leader-follower relation during a manipulation of the visual information available to musicians: musicians faced 180 degrees away from the center (to prevent direct visual contact with each other). They found a general decrease of communication across quartet members when they could not see each other, confirming that information flow is affected by a change in the available information. Extending these findings, Chang and colleagues^20^ quantified the intentionality of emotional expression using body sway kinematics of quartet musicians and Granger-Causality tools.

Beyond global descriptions of musician’s pattern of relationships, the complexity of these kinds of scenario could also be exploited to distinguish and evaluate the existence of multiple channels of communication as well as their respective role in efficient coordination. In previous studies, one representative kinematic parameter was used to extract global coordination^16,18–20^. However, we know that movements of different body parts may convey substantially different types of information. For instance, bow movements in violinists directly control the sound output (i.e., instrumental gestures), whereas complementary torso oscillations may serve a secondary communicative purpose (ancillary gestures)^15,21^. More importantly, movements of different body parts may act as different channels of communication, possibly with different roles depending on the specific communication mode. For example, within a quartet^18–20^, musicians have specific roles while in orchestras, musicians generally play in distinct sections (e.g. sections of violinists). This means that in the orchestra scenario, different modes of communication coexist: a complementary coordination with the conductor and other musicians, in parallel with an imitative coordination with musicians of the same group (playing the same score).

In the present study, we aim at answering two scientific questions: whether different channels of communication exist and whether they carry different information across modes of communication. We had a chamber orchestra playing music while we recorded bow and head kinematics (instrumental and ancillary movements) of a first and second section of violinists (four violinists in each section) as well as the arm and head kinematics of two different conductors. In one experimental condition we applied a perturbation to the orchestra sensorimotor information flow. The perturbation consisted in half-turn rotation of the first section of violinists so that they faced the second section and couldn’t see the conductor anymore. This perturbation modifies the perceptuo-motor context of the first section of violinists, placing also the second section and the conductor into a novel playing situation. By doing so, we analyzed inter-group complementary coordination as well as intra-group imitative coordination (modes of communication), through different channels of communication (instrumental and ancillary movements) during different playing situations (normal and perturbed).

Based on this experimental manipulation and, due to the new central role played by the second section (facing conductor and first section), we hypothesize a general increase of the influence of the second section on other musicians and conductor. At the same time, ensemble music playing require the co-regulation of the different modes of communication (e.g. cooperative/competitive)^22^ and adjustments in inter-group communication may be balanced by changes at the intra-group level. For instance, the additional efforts spent by the second section on inter-group communication may be at the expenses of intra-group coordination. We thus predict a decrease in the second section intra-group imitative coordination to focus on the communication with the first section and conductor. On the contrary, the first section may need to rely more on his own as would be shown by an increase intra-group imitative coordination. Finally, we predict that the two channels convey information that is differentially modulated across groups, modes and conditions. Specifically, information channeled through instrumental movements should be more robust to perturbations because they reflect overlearned patterns, which more closely relate to what is still available on the score. For this reason, we predict that bow movements may be less affected by the perturbation than the ancillary channel.

## Method

### Subjects

A chamber orchestra consisting of 8 violinists (2 sections of four violinists: S1 and S2) and 10 instrumentalists participated in the study along with two professional conductors (C1 and C2). Data were collected from the two violinists’ sections and conductors. Each violinists section counted four players. The study was approved by the SIEMPRE Project Management Committee and adhered to the standards laid down in the Declaration of Helsinki. All participants gave written informed consent before participating. The synchronized multimodal recordings of the musicians obtained for this experiment as well as the details of the SIEMPRE platform for multimodal recordings are made available to the research community from the EU ICT FET SIEMPRE web pages (http://www.siempre.infomus.org).

### Procedure

**Figure 1:**
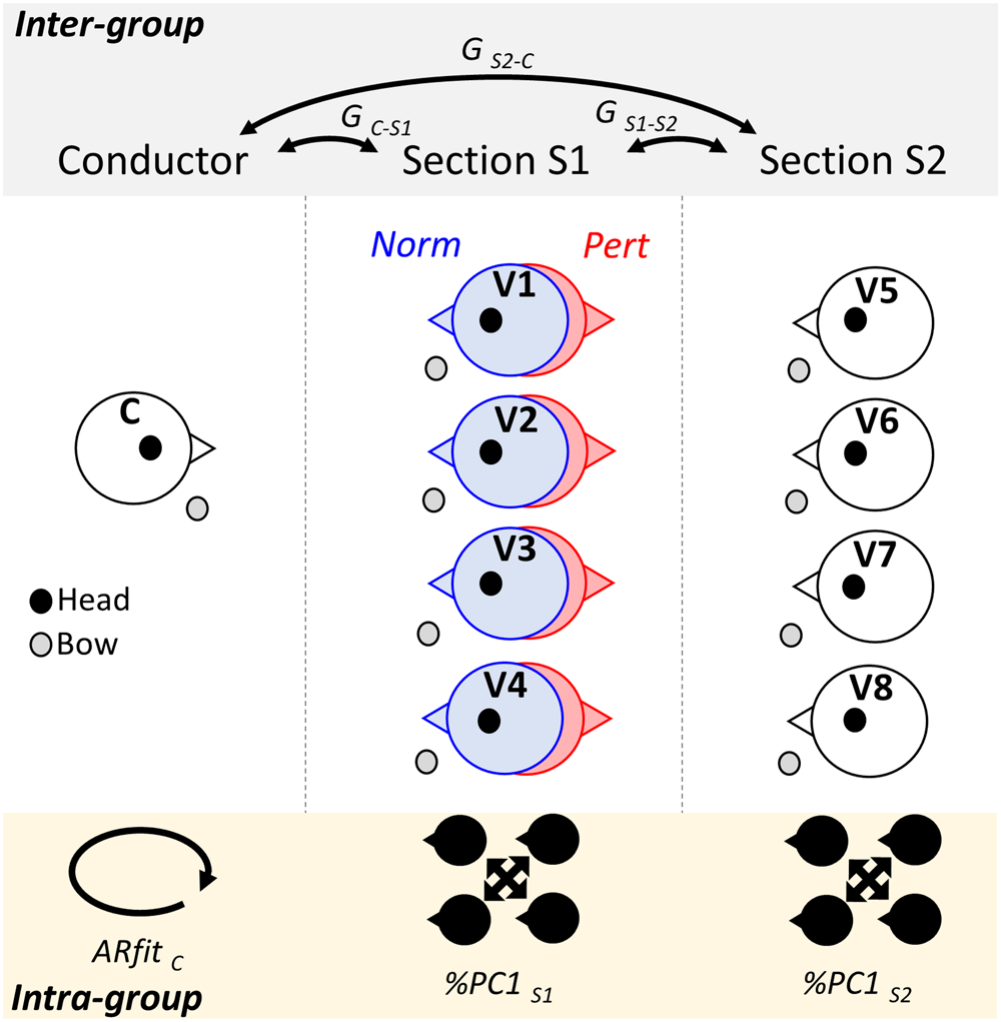
Orchestra musicians’ position and associated computations. The section in the middle represents the respective position of musicians in the orchestra: conductor (C), first section of violinists (S1; 4 violinists: V1, V2, V3 and V4) and second section of violinists (S2; 4 violinists: V5, V6, V7 and V8). In the normal condition (Norm; blue), S1 faces the conductor. In the perturbed condition (Pert; red), S1 rotates 180° facing S2. For each participant we recorded head (black dot) and bow (grey dot) kinematics. We extracted the pattern of communication at the group-level (between S1, S2 and C) using conditional Granger Causality (G) as shown in the top of the figure. Additionally, intra-group coordination, as described in the lower part of the figure, was computed via principal component analysis (%PC1) and an index of predictability of the conductor behavior (using the goodness of fit of the associated auto-regressive model; ARfit).

The two conductors and the orchestra executed two pieces of music selected from their repertoire so that their performance could already be at plateau and thus showing no learning during the experiment. The music pieces were excerpts from the ouverture of “Signor Bruschino” by Rossini and the Vivaldiana, terzo movimento by Malipiero (lasting around five minutes each). Two experimental conditions were tested (Fig. 1A), which only differ by the way one section (henceforth, first section, S1) interacts with the conductor and the other section (henceforth, second section, S2). In one condition (normal condition, Norm; Fig. 1 in blue), S1 violinists - lined in a single row - were able to see C, but not S2 violinists. This condition kept the standard position of the musicians. In the second condition (perturbed condition; Fig. 1 in red) S1 violinists - still lined in a single row - were able to see S2 violinists, but not C (since they were facing backwards with respect to him). This condition altered the standard position of the musicians. We decided to perturb S1 orientation to act on the strongest relationship (S1 - Conductor) and elicit a significant re-adaptation of the whole orchestra dynamics. By doing so, we could highlight the co-regulation of the different modes of communication, across different channels of communication. The two pieces were repeated six times (three times with C1 and three other times with C2) in each experimental condition (normal versus perturbed). In total, 24 trials were recorded.

### Apparatus and set-up

Movement data were collected (1000 Hz) by using a Qualisys motion capture system equipped with 7 cameras, integrated with the EyesWeb XMI platform: http://www.infomus.org/eyesweb_ita.php ^23^, including audio and physiological signals (not used here). Each violinist was equipped with a cap on which were placed three passive markers of the Qualisys motion capture system. The positions, based upon the 10–20 electroencephalographic system, corresponded to Pz, F3 and F4. Before starting recording sessions, we ensured that the cap was not moving with musicians’ movements and facial expressions. An average of these three markers was taken for further analysis on head movement, to minimize loss of data and interpolation. An additional marker was placed on the bows of the players and on the baton of the conductors. After data tracking by using the Qualysis Track Manager software, the data was exported and analyzed in MATLAB.

### Data pre-processing and analysis

#### Data pre-processing

We first used the spline method to handle the missing data in the 3D trajectories. The spline method interpolates missing data with continuous third order derivatives. We then computed the magnitude of the acceleration from each 3D trajectory (as done in)^16^. Acceleration was chosen because it should be more informative than trajectory and velocity, especially for what regards expressive information transfer. This claim is backed by studies on visuo-motor coordination suggesting that a marked deceleration towards the endpoint of a moving object’s trajectory provides more saliency to the timing of this endpoint, and facilitates synchronization with that object^24,25^. Each musician time-series on each trial was normalized (to z-scores) and outliers (>6std) were set as absent values (NaN) and interpolated when the gap was smaller than 200 frames (i.e. 2 sec). The total percentage of interpolated data was: 4.7% (±1.6).

In the following, we made an empirical dissociation between two modes of communications. We considered as intra-group imitative coordination, the relation between musicians playing the same score. Due to common score, these musicians are engaged in a joint behavior requiring an important degree of imitative coordination. In parallel, we named inter-group complementary coordination, the relation between musicians having different scores, and thus being engaged in a joint behavior requiring an important degree of movement complementarity.

#### Intra-section imitative coordination Principal Component Analysis

To evaluate the level of imitative coordination between violinists’ movements of each section of violinists (playing the same score), we used a principal component analysis (PCA)^26^. PCA is a standard statistical technique generally used to extract a low-dimensional structure from a high-dimensional dataset. Dimensionality reduction method are classically used in the motor synergies field to extract invariant/similar features across time between muscle or kinematic parameters. In particular, PCA has been used to characterize the degree of covariance across time of different body segments in whole-body movements (e.g. locomotion^27^; reaching)^28^. Here, PCA was performed on the acceleration profiles of the four violinists of each section (Fig. 1, lower panel), windowed and pre-processed in the same way as Granger Causality analysis. Mathematically, the method involves the eigenvalue decomposition of a dataset covariance matrix in order to find the principal directions in the high-dimensional space. For each of the windows, we considered an input matrix composed of 300 rows (temporal frames) and 4 columns (the acceleration profiles of the four violinists in each section) to which we applied the Matlab *princomp* function, after a zscore normalization of the input matrix. The PCA gives four principal components (PC) each written as a linear combination of the initial waveforms (the four violinists’ acceleration profile). The variance accounted for (VAF) by the first principal component (noted PC1%) is defined as the ratio between the first eigenvalue and the sum of all the eigenvalues. The VAF represents the degree to which the linear combination associated to each PC is able to approximate the initial dataset. A high PC1% value means that the trajectory in the space of angles is close to a straight line (i.e., all angles were linearly correlated together) while, a low PC1% value indicates that one principal component is not sufficient to describe precisely the trajectories.

#### Conductor behavior predictability: auto-regressive model’s fitting

Following sensorimotor communication literature (for a review)^29^, we evaluated conductor behavior predictability to verify whether intrinsic variability of conductor’s behavior was altered by our experimental manipulation. In cooperative joint action tasks, leaders tend to make their movements more consistent over time to help their partner build a predictive model of other’s action. An increase in the predictability of (Partner) A translates into a smaller uncertainty when (Partner) B needs to predict future signals coming from A to plan the most appropriate action. We evaluated the level of predictability of conductors’ behavior (Fig. 1, lower panel) as goodness of fit of the linear autoregressive model computed on the conductor acceleration profile extracted from bow and head data separately. We modelled the conductor acceleration profile via a linear autoregressive model in the same way we computed it for Granger Causality analysis and on the same sliding windows parameters. The optimal order of the model was determined via the Akaike’s information criterion and the goodness-of-fit (ARfit) was measured as the sum of squares of the residuals, for each sliding window.

#### Inter-group complementary coordination: Granger causality analysis

Granger causality analysis was then carried out on the preprocessed acceleration waveforms. According to Granger formalism, a signal X Granger-causes (or G-causes) a signal Y if the past values of X contains information that helps predict Y above and beyond the information contained in the past values of Y alone. Thus, a Granger-causality score (gca) was defined between each pair of musicians as the log-likelihood ratio of the degree to which the prior time series of a musician X (causing variable) contributes to predict the current status of a musician Y (dependent variable), over and above the degree to which it is predicted by its own prior time series while conditional on the remaining musicians time-series (conditional variables).

In our experimental context, the interaction of more than two time series was addressed. In this case, repeated pair-wise Granger causality computations can lead to misleading results. To avoid that, we used a simple extension of Granger causality, referred to as Conditional Granger causality^30^. Suppose we have three time series X, Y and Z, then the Conditional Granger causality from Y to X given Z is defined as the log ratio of the error variance of the restricted model where only Y is excluded from the history (when modeling X) and the variance of the unrestricted model, where the history of all time-series X, Y and Z is included. The use of conditional allow to take into account the influence of musicians out of the tested pair to avoid misinterpretation due to multiple sources of information^16,19^.

More details on Granger computation are reported in Supplementary Material.

Gca was evaluated (pairwise), every 500 milliseconds on 3-s sliding windows using the “Granger Causality connectivity analysis” Matlab toolbox^31^. Windows containing more than one third (i.e. 166 ms) of absent values were not used in the analysis (less than 5% of the total windows number). The Granger Causality computation is similar to the one used in^16,18^. From this point, we will represent gca of X on Y by the notations G_X->Y_ or X- > Y.

We were interested in the causality relations between the conductor and each section of violinists (S1 and S2). This analysis is illustrated in Fig. 1 (upper panel). We performed three different types of Conditional Granger causality computations: (1) Causality between each conductor and violinists of S1 (taken separately): defining as causing variable the conductor, as dependent variable each S1 violinist separately and the other way around [conditional variable: musicians in S2 - taken separately]. (2) Causality between each conductor and violinists of S2 (taken separately): defining as causing variable the conductor, as dependent variable each S2 violinist separately and the other way around [conditional variable: musicians in S1 - taken separately]. (3) Causality between the violinists of S1 and S2 (taken separately): defining as causing variable each S1 violinist separately, as dependent variable S2 violinists separately and the other way around [conditional variable: the conductor]. In these three analyses, we computed gca between each pair of musicians on each 3 s window. When the causality between the two variables was significant, we kept the gca value otherwise this value was set to 0. Finally, gca values were averaged across conditional variables, conductors and musicians of same section, to get one value per group (i.e. C- > S1, S1- > C, C- > S2, S2- > C, S1- > S2, S2- > S1). Thus, for each experimental condition, the output matrix consisted of 6 columns (the number of causal relation) and thousands of lines (the number of considered windows).

### Statistical analyses

Inter-group and intra-group data did not follow a normal distribution according to normality tests (Kolmogorov–Smirnov) and the variances were also not homogeneous according to statistical tests (Levene). We, therefore, used a two-tail independent samples Welch’s t-test (already used on same type of data in)^18^. In the Welch’s t-test the assumption of normality is not critical for large samples^32^ as it is the case for our data set. More importantly, Welch developed an approximation method for comparing the means of two independent populations when their variances are not necessarily equal^33^. Because Welch’s modified t-test is not derived under the assumption of equal variances, it allows the comparison of two populations without first having to test for equality of variance.

Based on the data extracted in “intra-section imitative coordination”, we made four comparisons for each kinematic parameter: %PC1_S1 NORM_ vs %PC1_S1 PERT_, %PC1_S2 NORM_ vs %PC1_S2 PERT_, %PC1_S1 NORM_ vs %PC1_S2 NORM_, %PC1_S1 PERT_ vs %PC1_S2 PERT_. For “conductor behavior predictability” we compared for each kinematic parameter: ARfit _NORM_ vs ARfit _PERT_.

Based on the data extracted in the “inter group complementary coordination”, we made three different set of comparisons, repeated twice (once for head data, once for bow data). (1) For the normal condition, we ran 5 comparisons: C- > S1 vs S1- > C, C- > S2 vs S2- > C, S1- > S2 vs S2- > S1, C- > S1 vs C- > S2, S1- > C vs S2- > C. The other possible comparisons were not performed because they were not informative for the study (e.g. C- > S1 vs S2- > C) or comparing elements of different nature (e.g. C- > S1 vs S2- > S1). (2) For the perturbed condition, we ran the same 5 comparisons as in (1). (3) Across the two experimental conditions, we ran 6 comparisons: C- > S1_NORM_ vs C- > S1_PERT_, C- > S2_NORM_ vs C- > S2_PERT_, S1- > C_NORM_ vs S1- > C_PERT_, S2- > C_NORM_ vs S2- > C_PERT_, S1- > S2_NORM_ vs S1- > S2_PERT,_ S2- > S1_NORM_ vs S2- > S1_PERT_.

In all these analyses, the p-level was corrected for multiple comparisons with the Benjamini and Hochberg false discovery rate procedure. We reported in the results part the corrected p-value, and the value of the test statistic. We considered as marginally significant the statistical comparison for which the p-value before correction was inferior to 0.05. All analyses were conducted using the Matlab Statistics toolbox (Mathworks Inc.).

## Results

### Intra-section imitative coordination (Principal component analysis)

#### Bow data

**Figure 2:**
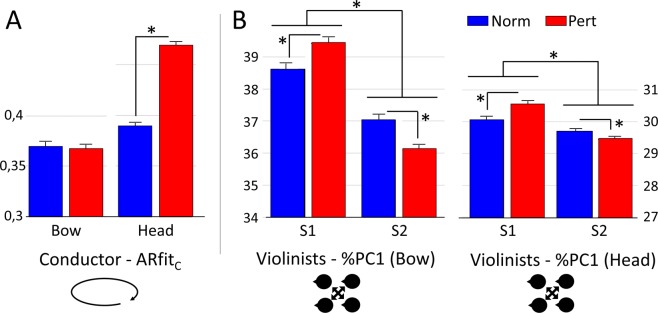
(**A**) Predictability of the conductor behavior (ARfit). The autoregressive model was computed on the conductors, from bow and head data separately for the two experimental conditions normal (black) and perturbed (white). (**B**) Intra-section synchronization as indexed by the percentage of reconstruction of the first principal component (%PC1). PCA analysis was run on bow (middle panel) and head (right panel) acceleration profiles of the four violinists of each section (S1 and S2). Statistical differences are represented by black lines on the top of each histogram.

The %PC1 increased from Norm to Pert for S1 (Fig. 2B, left panel; p < 0.01; t = −3.22) while decreased for S2 (p < 0.001; t = 4.03). In addition, %PC1 was larger for S1 compared to S2 in the two experimental conditions (Norm: p < 0.001; t = 7.11; Pert: p < 0.001; t = 12.97).

#### Head data

**Figure 3:**
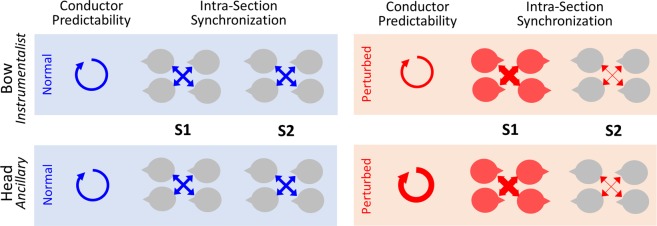
Schematic representation of the main results for intra-group analysis. Results associated to the two channels, bow and head, are displayed respectively in the upper and lower panel. Circular arrows displayed in the left panel represent the strength of conductor predictability (ARfit). Middle and right panels represent the intra-group synchronization’s (%PC1) for both sections of violinists. Thickness of the arrow represent the strength of the effect in each experimental condition: normal (blue) and perturbed (red).

A similar pattern of results was found for head data. The %PC1 increased from Norm to Pert for S1 (Fig. 2B, right panel; p < 0.001; t = −5.35) while decreased for S2 (p < 0.01; t = 3.25). In addition, %PC1 was larger for S1 compared to S2 in the two experimental conditions (Norm: p < 0.001; t = 4.42; DoF = 5450; Pert: p < 0.001; t = 12.03). An illustrated version of these results is presented in Fig. 3.

### Conductor behavior predictability (auto-regressive model’s fitting)

#### Bow data

The goodness of fit of the autoregressive model for the bow data was not different in the two conditions Norm and Pert (p = 0.81; t: 0.25; Fig. 2A).

#### Head data

The goodness of fit of the autoregressive model for the head data was significantly smaller in Norm compared to Pert (p < 0.001; t = −14.95; Fig. 2A). An illustrated version of these results is presented in Fig. 3.

### Inter-group complementary coordination (Granger causality analysis)

#### Bow data

**Figure 4:**
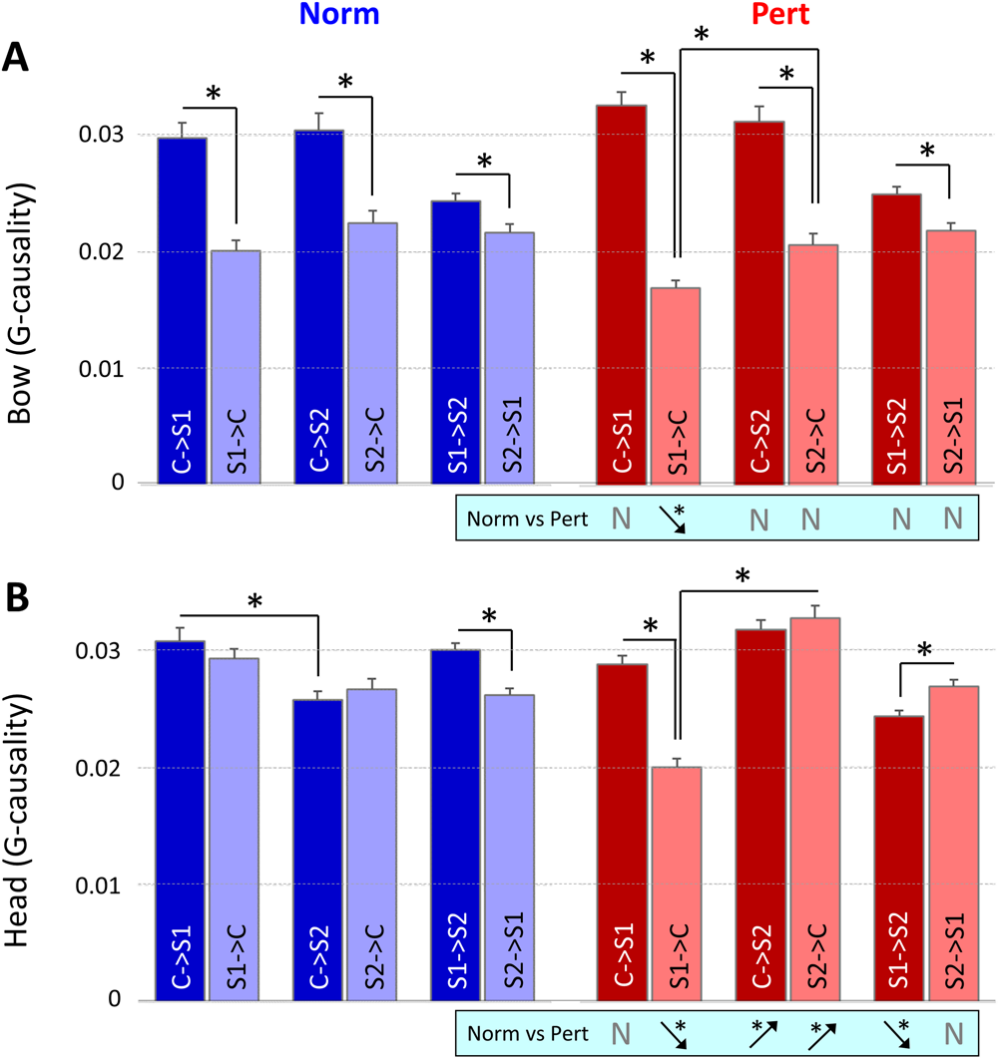
Inter-group coordination (gca). Values extracted from Bow (**A**) and Head (**B**) acceleration profiles are shown for the normal (left side) and perturbed conditions (right side). Statistical differences within each condition are marked by colored lines on the top of each histogram. Statistical differences between conditions (Norm vs Pert) are represented by black lines under each histogram.

(1) In the normal condition, C G-caused S1 and S2 more than the other way around (Fig. 4A, left panel; C < - > S1: p < 0.001, t = 6.08; C < - > S2: p < 0.001, t = 4.34). The gca of S1 on S2 was larger than the gca of S2 on S1 (p < 0.05, t = 2.63). No other significant differences appeared in Norm. (2) The pattern was the same in the perturbed condition (Fig. 4A, right panel). C G-caused S1 and S2 more than the other way around (C < - > S1: p < 0.001, t = 11.55; C < - > S2: p < 0.001, t = 6.72). The gca of S1 on S2 was larger than the gca of S2 on S1 (p < 0.01; t = 3.31). In addition, the gca of S1 on the conductor was significantly smaller than the gca of S2 on the conductor (p < 0.01, t = −3.17). No other significant differences appeared in Perturbed. (3) A significant decrease from Norm to Pert appeared in the gca of S1 on C (p < 0.05, t = 2.73). We found no additional significant change between the two conditions (Fig. 4A, lower blue rectangle).

#### Head data

(1) In the normal condition, no significant difference appeared between the gca of C on S1 and S2 compared to the inverse relation (Fig. 4B, left panel; C < - > S1: p = 0.29; C < - > S2: p = 0.40). A significant difference was found between the gca of S1 on S2 and S2 on S1: G_S1->S2_ being higher than G_S2->S1_ (p < 0.001; t = 3.72). In addition, the gca of C on S1 was larger than C on S2 (p < 0.001; t = 3.69). No other significant difference appeared in Norm. (2) In the perturbed condition (Fig. 4B, right panel), C G-caused S1 significantly more than the inverse (p < 0.001; t = 5.64). In addition, the significant difference between the gca of S1 on S2 and S2 on S1 changed of direction compared to Norm: G_S1->S2_ being smaller than G_S2->S1_ (p < 0.01; t = −2.69). Additionally, the gca of S2 on C was larger than the one of S1 on C (p < 0.001; t = −7.89). No other significant differences appeared in Pert. (3) Comparing the two experimental conditions (Fig. 4B, lower blue rectangle), we found a significant increase of G_C->S2_ (p < 0.001; t = −4.32) and G_S2->C_ (p < 0.001; t = −3.87) and a significant decrease of G_S1->C_ (p < 0.001; t = 6.47) and G_S1->S2_ (p < 0.001; t = 5.90) in Pert compared to Norm.

**Figure 5:**
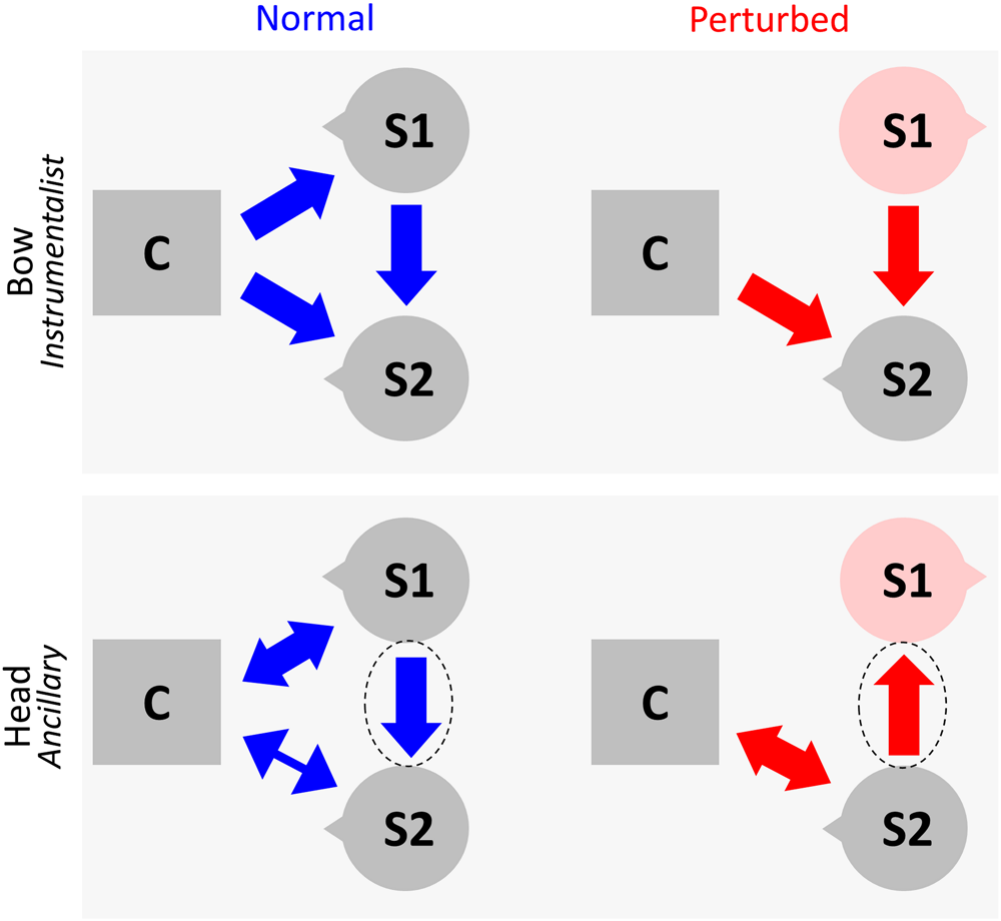
Schematic representation of the main results for inter-group Granger-Causality analysis (i.e. inter-group coordination) across the conductor (C) and the two sections of violinists (S1 and S2). Results associated to the two channels, bow and head, are displayed respectively in the upper and lower panel. Directional arrows illustrate inter-group coordination (C, S1 and S2), in the normal (blue) and perturbed (red) condition. Arrows thickness represents the interaction’s strength. A bidirectional arrow indicates similar gca values for the two directions (i.e. group 1 G-causes group 2, as much as group 2 G-causes group 1). On the opposite, a unidirectional arrow indicates the direction of the larger gca value (e.g. group 1 G-causes more group 2, than the inverse). To highlight the difference between the two conditions, we did not represent the arrow between C and S1.

An illustrated version of these results is presented in Fig. 5.

## Discussion

Social interaction requires mastering the integration of multimodal sources of information to achieve efficient interpersonal coordination. Behavioral adaptation and synchronization are fundamentally based on predictive mechanisms and on the ability to use previous experience and context to guide perceptual processes while interaction unfolds^34^. Recently, an important resurgence of interest has emerged towards the exploration of human cognition in its true context, which is fundamentally interactive^35,36^. Within this stream, ensemble musicians have been described as a powerful model to investigate complex non-verbal communication^14,15^.

The analysis of multi-agent kinematics via the Granger Causality method has shown important promise^16^. For instance, in orchestra, this method allowed the extraction of group-level information flow^16^ which is associated to the quality of the musical output. Furthermore, by applying perturbations to the communication flow in quartets, subsequent studies showed rapid ensemble adaptation to sensorimotor information exchange^18,19^. The present study tackled two scientific questions that had not been explored in previous experiments: whether different channels of communication exist (as mediated by ancillary Vs. instrumental movements) and whether they carry information that is critical for different modes of ensemble coordination (e.g. inter-group complementary and intra-group imitative coordination).

Regarding the different channels of communication, successful interaction generally requires that participants send and receive subtle messages in the form of various motor gestures. Musician’s movements can be separated into instrumental and ancillary. In violinists, upper limbs movements are directly linked to the production of music while head and trunk oscillation may carry additional information at the phrase level^37,38^. For instance, subjective evaluation of conductors’ face movements were rated higher in expressivity, whereas arms movements were judged higher in amount of musical information^39^. Notably, few studies could dissociate these different channels empirically in the context of a joint musical performance^40^.

Our results demonstrate that the pattern of sensorimotor information carried by two selected movements (head and bow) are distinct. Bow kinematics exhibit a robust leader-follower relationship between the conductor and the two violinists’ sections. This pattern is substantially not affected by the experimental manipulation of the sensorimotor information flow (perturbed condition) except for a decrease in communication between the first section and the conductor. The fact that the perturbation did not dramatically alter the information exchanged via instrumental movements suggests an important role of memory, score reading and residual sensory cues. Indeed, musicians train for several hours and may rely on rehearsal memory to cope with the perturbation, at least for what concern pure instrumental execution. At the same time, there is also a clear directionality of the information flow from conductor to musicians, which confirms the idea of a predominant role of the conductor in the group management^41^.

Ancillary movements, instead, are supposed to convey slower frequency signals possibly related to the expressive component of musical execution, which is more likely to be affected by perturbation of the interaction dynamics. In fact, in head data, the perturbation produced clear alteration of the communication pattern. Communication between the first section and the conductor or the second section was reduced. At the same time, communication between the second section and the conductor increased in both directions. This global increase suggests a greater need for information exchange during the perturbation and considering that conductors and S2 did not change their positions, we observe a quantitative but not a qualitative alteration of their communication. Instead, moving to the relationship between S1 and S2 we observe a complete reversal of their mutual communication. Before the perturbation, the first section provided larger causal drive towards the second section, while after, the second section lead the first. During the perturbation, the first section no longer had visual contact with the conductor, significantly reducing his role in leading orchestra dynamics. Even if we cannot exclude the contribution of haptic, acoustic or residual visual information, the remaining influence that the conductor exert on S1, seems to be mediated by the new role played by S2. Although violinists of the second section did not actually change their position, they are the only ones establishing direct face-to-face communication with both first section and conductor. Interestingly, they seem to increase their normal communication with conductors, while at the same time they dramatically change the way the communicate with S1. Correspondingly, our results suggest that S2 musicians were implicitly invested with far more centrality in orchestra coordination dynamics.

The distinct modulation of head versus bow kinematic parameters provides a demonstration of the multi-level complexity of musicians’ coordination. At the same time, another important aspect is related to the co-regulation of different modes of interaction. In our experimental context, each violinist must exchange information with other musicians of the same section (playing the same musical score – intra-group imitative coordination) and with other participants (playing different parts – inter-group complementary coordination). We used PCA to complement inter-group Gca analysis with an estimation of intra-section imitative coordination. Both kinematic parameters highlighted similar pattern of results. Due to the lack of communication with the conductor, the first section became more coordinated, in the probable attempt to maximize performance accuracy. On the contrary, the second section that was endowed with the central role of being the communication hub, reduced intra-group coordination. This may be driven by a need to gain the necessary degrees of freedom to lead communication with S1 and be the sole interlocutor of the conductor. Therefore, here we show that to modulate inter-group dynamics, S2 violinists had to penalize imitative coordination at the intra-group level.

Similar co-regulations of different modes of communication have already been described. For instance, a mixture of cooperative and competitive behaviors in choir singing^22^ and a balance between self-other integration and segregation in joint piano playing^42^ was shown earlier. In general, any complex human interaction may require a mixture of temporal coordination and imitation^43^ together with the coordination of complementary actions^8^. Although these two modes of interaction may naturally co-occur, it is difficult to explore them together in an experimentally controlled environment. Using the specificity of the orchestra scenario we explored here the interaction of intra-group dynamics (dominated by imitative coordinated behaviors) and inter-group dynamics (characterized by complementary action coordination).

Finally, we found an increase in conductor predictability following the perturbation, on head data only. Increasing behavioral predictability is a signaling strategy already described for leaders in dyadic interaction^29,44,45^. In the musical domain, Wöllner and colleagues^46^ showed that musicians could better synchronize with a “morphed” virtual conductor (made by averaging the movements of multiple conductors) compared to individual conductors. Indeed, morphed conductor may provide a more prototypical example (with minimal noise), allowing a greater readability and thus facilitating temporal prediction. In this sense, greater behavioral consistency may be a key implicit coordination strategy to help musicians build a more reliable internal predictive model of the task and conductors’ movements.

In fact, internal predictive models, built through practice and previous experiences, may be essential in guiding individual action into an efficient coordination with peers^1,2,47,48^. Behavioral predictions are confronted with sensorial feedbacks^49,50^ and may help to coordinate actions requiring fast and precise temporal coordination^51^, in which information can be sampled only intermittently. In professional musicians, extensive training may allow the construction of a detailed model of the piece and associated interactions. This model may, in turn, allow a good performance even in sub-optimal conditions, such as the one designed here. Indeed, extensive musical training has been associated with anatomo-functional changes^52,53^ paralleled by enhanced ability to discriminate subtle changes in others’ performance via predictive action simulation^54,55^.

In conclusion, our work highlights the multidimensionality of group coordination by evidencing different channels of communication (ancillary versus instrumental movements), affecting coordination at different levels (inter-group versus intra-group) tapping into different modes of cooperation (complementary versus imitative coordination). The co-regulation of these elements is the key musicians use to flexibly adapt to perturbation of the normal information flow and that is potentially shared with other non-musical complex ecological interaction.

### Limitations

It is important to note that the current study explored naturalistic behaviors at the cost of having less experimental control^15^. Due to the complexity of the data collection and the necessarily limited number of times one can ask musicians to play the same piece, we decided to apply only one perturbation. Perturbing the strongest relationship (S1 - Conductor) allowed us to elicit a significant re-adaptation of the whole orchestra dynamics across the different modes and channels of communication. Future research will have to expand the current investigation along several directions. For instance, remains to be seen whether perturbing S2 produces similar important adaptation of orchestra dynamics. At the same time, the analysis of arm and head kinematics, although theoretically motivated^15^, provides only a relatively limited view about the complexity of group dynamics. For instance, the inclusion of physiological signals such as electromyography, heart rate or galvanic skin responses may open to the affective dimension of music ensemble coordination.

## Supplementary information

Details of Granger Causality Analysis

## References

[1] Wolpert DM, Doya K, Kawato M (2003). A unifying computational framework for motor control and social interaction. Philos. Trans. R. Soc. London.

[2] Sebanz N, Knoblich G (2009). Prediction in Joint Action: What, When, and Where. Top. Cogn. Sci..

[3] Jeannerod M (2001). Neural simulation of action: a unifying mechanism for motor cognition. Neuroimage.

[4] Friston KJ, Mattout J, Kilner JM (2011). Action understanding and active inference. Biol. Cybern..

[5] van Ulzen NR, Lamoth CJC, Daffertshofer A, Semin GR, Beek PJ (2008). Characteristics of instructed and uninstructed interpersonal coordination while walking side-by-side. Neurosci. Lett..

[6] Richardson MJ, Marsh KL, Isenhower RW, Goodman JRL, Schmidt RC (2007). Rocking together: Dynamics of intentional and unintentional interpersonal coordination. Hum. Mov. Sci..

[7] Newman-Norlund, R. D., Noordzij, M. L., Meulenbroek, R. & Bekkering, H. Exploring the brain basis of joint action: Co-ordination of actions, goals and intentions. *Soc. Neurosci*. **2** (2007). 10.1080/17470910701224623 18633806

[8] Sebanz N, Bekkering H, Knoblich G (2006). Joint action: Bodies and minds moving together. Trends Cogn. Sci..

[9] Fessler DMT, Holbrook C (2016). Synchronized behavior increases assessments of the formidability and cohesion of coalitions. Evol. Hum. Behav..

[10] Dikker S (2017). Brain-to-Brain Synchrony Tracks Real-World Dynamic Group Interactions in the Classroom. Curr. Biol..

[11] Alderisio, F., Fiore, G., Salesse, R. N., Bardy, B. G. & di Bernardo, M. Interaction patterns and individual dynamics shape the way we move in synchrony. *arXiv* at, http://arxiv.org/abs/1607.02175 (2016). 10.1038/s41598-017-06559-4 PMC5533803 28754908

[12] Codrons, E., Bernardi, N. F., Vandoni, M. & Bernardi, L. Spontaneous group synchronization of movements and respiratory rhythms. *PLoS One* **9** (2014). 10.1371/journal.pone.0107538 PMC4162643 25216280

[13] McNeill, D. *Language and Gesture*. (2000).

[14] Volpe G, D’Ausilio A, Badino L, Camurri A, Fadiga L (2016). Measuring social interaction in music ensembles. Philos. Trans. B.

[15] D’Ausilio A, Novembre G, Fadiga L, Keller PE (2015). What can music tell us about social interaction?. Trends Cogn. Sci..

[16] D'Ausilio Alessandro, Badino Leonardo, Li Yi, Tokay Sera, Craighero Laila, Canto Rosario, Aloimonos Yiannis, Fadiga Luciano (2012). Leadership in Orchestra Emerges from the Causal Relationships of Movement Kinematics. PLoS ONE.

[17] Colley ID, Varlet M, MacRitchie J, Keller PE (2018). The influence of visual cues on temporal anticipation and movement synchronization with musical sequences. Acta Psychol. (Amst)..

[18] Badino L, D’Ausilio A, Glowinski D, Camurri A, Fadiga L (2014). Sensorimotor communication in professional quartets. Neuropsychologia.

[19] Chang A, Livingstone SR, Bosnyak DJ, Trainor LJ (2017). Body sway reflects leadership in joint music performance. Proc. Natl. Acad. Sci..

[20] Chang, A., Kragness, H. E., Livingstone, S. R., Bosnyak, D. J. & Trainor, L. J. Body sway reflects joint emotional expression in music ensemble performance. *Sci. Rep*. **9** (2019). 10.1038/s41598-018-36358-4 PMC6338747 30659220

[21] Benbasat Ari Y., Paradiso Joseph A. (2002). An Inertial Measurement Framework for Gesture Recognition and Applications. Gesture and Sign Language in Human-Computer Interaction.

[22] Keller PE, König R, Novembre G (2017). Simultaneous cooperation and competition in the evolution of musical behavior: Sex-Related modulations of the singer’s formant in human chorusing. Front. Psychol..

[23] Volpe, G. *et al*. Designing multimodal interactive systems using eyesweb xmi. In *Proceedings of the First International Workshop on Smart Ecosystems cReation by Visual design co-located with the International Working Conference on Advanced Visual Interfaces* 49–56 (2016).

[24] Varlet M (2014). Difficulty leading interpersonal coordination: towards an embodied signature of social anxiety disorder. Front. Behav. Neurosci..

[25] Zelic G, Varlet M, Kim J, Davis C (2016). Influence of pacer continuity on continuous and discontinuous visuo-motor synchronisation. Acta Psychol. (Amst)..

[26] Jolliffe IT (2002). Principal Component Analysis, Second Edition. Encycl. Stat. Behav. Sci..

[27] Hicheur Halim, Terekhov Alexander V., Berthoz Alain (2006). Intersegmental Coordination During Human Locomotion: Does Planar Covariation of Elevation Angles Reflect Central Constraints?. Journal of Neurophysiology.

[28] Berret B, Bonnetblanc F, Papaxanthis C, Pozzo T (2009). Modular Control of Pointing beyond Arm’s Length. J. Neurosci..

[29] Pezzulo Giovanni, Donnarumma Francesco, Dindo Haris, D'Ausilio Alessandro, Konvalinka Ivana, Castelfranchi Cristiano (2019). The body talks: Sensorimotor communication and its brain and kinematic signatures. Physics of Life Reviews.

[30] Ding, M., Chen, Y. & Bressler, S. L. *Handbook of Time Series Analysis. Wiley* (2006).

[31] Seth AK (2010). A MATLAB toolbox for Granger causal connectivity analysis. J. Neurosci. Methods.

[32] Geary RC (1947). Testing for Normality. Biometrika.

[33] Welch BL (1947). The generalization of ‘Student’s’ problem when several different population variances are involved. Biometrika.

[34] Donnarumma F, Costantini M, Ambrosini E, Friston KJ, Pezzulo G (2017). Action perception as hypothesis testing. Cortex.

[35] Schilbach L (2013). Toward a second-person neuroscience. Behav. Brain Sci..

[36] Hari R, Henriksson L, Malinen S, Parkkonen L (2015). Centrality of Social Interaction in Human Brain Function. Neuron.

[37] Poggi, I. Music and leadership, the choir conductor’s multimodal communication. *Integr. Gestures* 341–353 (2011).

[38] Gritten, A. & King, E. *New perspectives on music and gesture*. (2011).

[39] Wöllner C (2008). Which part of the conductor’s body conveys most expressive information? A spatial occlusion approach. Music. Sci..

[40] Ragert M, Schroeder T, Keller PE (2013). Knowing too little or too much: The effects of familiarity with a co-performer’s part on interpersonal coordination in musical ensembles. Front. Psychol..

[41] Atik Y (1994). The Conductor and the Orchestra: Interactive Aspects of the Leadership Process. Leadersh. Organ. Dev. J..

[42] Novembre G, Sammler D, Keller PE (2016). Neural alpha oscillations index the balance between self-other integration and segregation in real-time joint action. Neuropsychologia.

[43] Keller PE, Novembre G, Hove MJ (2014). Rhythm in joint action: psychological and neurophysiological mechanisms for real-time interpersonal coordination. Philos. Trans. R. Soc. Lond. B. Biol. Sci..

[44] D’Ausilio A (2015). Automatic imitation of the arm kinematic profile in interacting partners. Cogn. Process..

[45] Vesper C, Van Der Wel RPRD, Knoblich G, Sebanz N (2011). Making oneself predictable: Reduced temporal variability facilitates joint action coordination. Exp. Brain Res..

[46] Wöllner C, Deconinck FJA, Parkinson J, Hove MJ, Keller PE (2012). The perception of prototypical motion: Synchronization is enhanced with quantitatively morphed gestures of musical conductors. J. Exp. Psychol. Hum. Percept. Perform..

[47] Novembre G, Ticini LF, Schütz-Bosbach S, Keller PE (2014). Motor simulation and the coordination of self and other in real-time joint action. Soc. Cogn. Affect. Neurosci..

[48] Ramnani N, Miall RC (2004). A system in the human brain for predicting the actions of others. Nat. Neurosci..

[49] Kilner JM, Frith CD (2007). Predictive coding: an account of the mirror neuron system. Cogn. Process..

[50] Friston KJ, Kilner JM, Harrison L (2006). A free energy principle for the brain. J. Physiol..

[51] Knoblich, G., Butterfill, S. & Sebanz, N. *Psychological Research on Joint Action. Theory and Data*. *Psychology of Learning and Motivation - Advances in Research and Theory* **54** (2011).

[52] Münte TF, Altenmüller E, Jäncke L (2002). The musician&apos;s brain as a model of neuroplasticity. Nat. Rev. Neurosci..

[53] Zatorre RJ, Chen JL, Penhune VB (2007). When the brain plays music: auditory-motor interactions in music perception and production. Nat. Rev. Neurosci..

[54] D’Ausilio A, Brunetti R, Delogu F, Santonico C, Belardinelli OM (2010). How and when auditory action effects impair motor performance. Exp. Brain Res..

[55] Candidi M, Sacheli LM, Mega I, Aglioti SM (2014). Somatotopic mapping of piano fingering errors in sensorimotor experts: TMS studies in pianists and visually trained musically naïves. Cereb. Cortex.

