# Author Correction: Multi-layer adaptation of group coordination in musical ensembles

**DOI:** 10.1038/s41598-019-55965-3

**Published:** 2020-01-14

**Authors:** Pauline M. Hilt, Leonardo Badino, Alessandro D’Ausilio, Gualtiero Volpe, Serâ Tokay, Luciano Fadiga, Antonio Camurri

**Affiliations:** 1 IIT @UniFe Center for Translational Neurophysiology of Speech and Communication, Istituto Italiano di Tecnologia, Ferrara, Italy; 2 Section of Human Physiology, Università di Ferrara, Ferrara, 44121 Italy; 3 Casa Paganini—InfoMus, DIBRIS, Universita degli Studi di Genova, Viale Causa 13, Genova, 16145 Italy; 4 Philarmonie de Chambre Tokay, Paris, France

Correction to: *Scientific Reports* 10.1038/s41598-019-42395-4, published online 10 April 2019

The original version of this Article contained errors in the names of the authors Pauline M Hilt, Leonardo Badino, Alessandro D’Ausilio, Gualtiero Volpe, Serâ Tokay, Luciano Fadiga, Antonio Camurri which were incorrectly given as Hilt Pauline M, Badino Leonardo, D’Ausilio Alessandro, Volpe Gualtiero, Tokay Serâ, Fadiga Luciano, and Camurri Antonio

In addition, this Article contained errors in the Acknowledgements section.

“This work was supported by Min. Salute Ric. Finalizzata 2016 - Giovani Ricercatori to AD; POETICON++ FP7-ICT-288382 and EnTimeMent H2020-FETPROACT-824160 to LF.”

now reads:

“This work was supported by Min. Salute Ric. Finalizzata 2016 - Giovani Ricercatori to AD; POETICON++ FP7-ICT-288382 to LF; SIEMPRE FP7-ICT-250026 and EnTimeMent H2020-FETPROACT-824160 to AC and LF.”

These errors have now been corrected in the HTML and PDF versions of the Article, and in the accompanying Supplementary Material.

