# Supplementary material for: Multi-layer adaptation of group coordination in musical ensembles
Source: Sci Rep. 2019 Apr 10;9:5854. doi: 10.1038/s41598-019-42395-4 (PMC6458170; doi:10.1038/s41598-019-42395-4)
Supplement: Supplementary file 1 — Details of Granger Causality Analysis [file 41598_2019_42395_MOESM1_ESM.pdf]

**Multi-layer adaptation of group coordination in musical ensembles**

Hilt[1] PM, Badino[1] L, D'Ausilio[1,2] A, Volpe[3] G, Tokay[4] S, Fadiga[1,2] L, and Camurri[3] A

## *Granger Causality*

Granger causality, in its standard and linear formulation, is based on (linear) Autoregressive Models (AR). AR models belong to the family of the Linear Dynamical Systems, which has been extensively used in modeling human motion (Bissacco, 2005; Del Vecchio, Murray, & Perona, 2003; Lu & Ferrier, 2004). An AR(k) model of a time series $x$ is defined as:

$$(1) \quad x(t) = \sum_{j=1}^{l} a_j x(t-j) \; + \; \varepsilon_R(t)$$

where $x(t)$ is the value of the time series $x$ at time $t$, $l$ is the order of the model (i.e., the length of the history observed in the model), $a_j$ $(j = 1, \ldots, l)$ are the weights for the history (the model parameters), and $\varepsilon_R(t)$ is the residual (prediction error).

There are two widely used criteria for selecting the optimal order of a linear predictor (i.e., the order that guarantees the best goodness of fit of the model, sometimes referred to as history): the Akaike's Information Criterion (AIC) (Akaike, 1974) and the Schwarz's Bayesian Information Criterion (BIC) (Schwartz, 1978). The parameters $a_j$ can be computed by using Ordinary Least Squares.

Since Granger causality is based on AR models the validity of the inferred causal relations depends on the validity of the AR models (more specifically of the unrestricted AR models, see next section). To assess the validity of an AR model different tests can be carried out. We evaluated the goodness-of-fit of the model (measured as the sum of squares of the residuals).

Granger formalism: A time series X is said to "Granger cause" a time series Y, if the past values of X provide statistically significant information to predict the next value of Y (Granger, 1969). The prediction is computed using AR models. Two AR models are required: an unrestricted AR model where the history of all time series is assumed to contribute to the prediction of the current value of a time series; and a restricted AR model where the time series whose causality value (on the other time series) is computed is excluded from the history. Given two time series X and Y, the unrestricted model is defined as:

$$x(t) = \sum_{j=1}^{l} a_{U,j} x(t-j) + \sum_{j=1}^{l} b_{U,j} y(t-j) + \varepsilon_U(t)$$

(2)

$$y(t) = \sum_{j=1}^{l} c_{U,j} x(t-j) + \sum_{j=1}^{l} d_{U,j} y(t-j) + \eta_U(t)$$

While the restricted model is defined as:

$$x(t) = \sum_{j=1}^{l} a_{R,j} x(t-j) + \varepsilon_R(t)$$

(3)

$$y(t) = \sum_{j=1}^{l} d_{R,j} y(t-j) + \eta_R(t)$$

Then the magnitude of the causality from X to Y and from Y to X can be measured respectively as:

(4) $$\mathcal{F}_{x\to y} = \ln\frac{\mathrm{H}_R}{\mathrm{H}_U}, \qquad \mathcal{F}_{y\to x} = \ln\frac{\mathrm{E}_R}{\mathrm{E}_U}$$

where E and H are the model error variances:

(5) $$\mathrm{E}_R = var(\varepsilon_R(t)), \ \mathrm{E}_U = var(\varepsilon_U(t)),$$

$$\mathrm{H}_R = var(\eta_R(t)), \ \mathrm{H}_U = var(\eta_U(t))$$

Once the Granger causality values have been computed, we need to test their statistical significance, i.e., we need to infer the significant causal relations. A significance test was done by carrying out an F-test of the null hypothesis that the model parameters referring to the time series of which we compute the “causal strength” (on the other time series) are all zero (e.g., parameters $b_{U,j}$ in model (2) to test the significance of $\mathcal{F}_{y\to x}$ ). When more than two time series are analyzed some corrections (here, the Bonferroni correction) were applied to the F-test.

In our experimental context, the interaction of more than two time series was addressed. In this case, repeated pair-wise Granger causality computations can lead to misleading results. To avoid that, we used a simple extension of Granger causality, referred to as Conditional Granger causality, (Ding, Chen, & Bressler, 2006). Suppose we have three time series X, Y and Z, then the Conditional Granger causality from Y to X given Z is defined as the log ratio of the error variance of the restricted model where only Y is excluded from the history (when modeling X) and the variance of the unrestricted model, where the history of all time series X, Y and Z is included.

The Granger causality analysis, including AR model validation and statistical tests of causal interactions, were carried out by using the “Granger Causality Connectivity Analysis” Matlab toolbox.

Based on Conditional Granger causality, we computed gca value between each pair of musicians every 500 milliseconds on 3-s sliding windows. For each 3s window, when the causality between the

two variables was significant, we kept the gca value otherwise this value was set to 0. Finally, these values were averaged across conditional variables, conductors and musicians of same section, to get one value per group (i.e. C->S1, S1->C, C->S2, S2->C, S1->S2, S2->S1). Thus, for each experimental condition, the output matrix consisted of 6 columns (the number of causal relation) and thousands of lines (the number of considered windows).

***References***


Akaike, H. (1974). A new look at the statistical model identification. In *IEEE Trans. Autom. Control* (pp. 716–723).

Ancona, N., Marinazzo, D., & Stramaglia, S. (2004). Radial basis function approach to nonlinear Granger causality of time series. *Physical Review E - Statistical Physics, Plasmas, Fluids, and Related Interdisciplinary Topics*, *70*(5), 7. http://doi.org/10.1103/PhysRevE.70.056221

Bissacco, A. (2005). Modeling and learning contact dynamics in human motion. In *IEEE Computer Society Conference on Computer Vision and Pattern Recognition CVPR05* (pp. 421–428). http://doi.org/10.1109/CVPR.2005.225

Chen, Y., Rangarajan, G., Feng, J., & Ding, M. (2004). Analyzing multiple nonlinear time series with extended Granger causality. *Physics Letters, Section A: General, Atomic and Solid State Physics*, *324*(1), 26–35. http://doi.org/10.1016/j.physleta.2004.02.032

Del Vecchio, D., Murray, R. M., & Perona, P. (2003). Segmentation of human motion into dynamics based primitives with application to drawing tasks. *Automatica*, *39*, 1–36. http://doi.org/10.1109/ACC.2003.1239860

Ding, M., Chen, Y., & Bressler, S. L. (2006). *Handbook of Time Series Analysis*. (S. Schelter, N. Winterhalder, & J. Timmer, Eds.)*Wiley* (Wiley). Wienheim.

Freiwald, W. A., Valdes, P., Bosch, J., Biscay, R., Jimenez, J. C., Rodriguez, L. M., … Singer, W. (1999). Testing non-linearity and directedness of interactions between neural groups in the macaque inferotemporal cortex. *Journal of Neuroscience Methods*, *94*(1), 105–119. http://doi.org/10.1016/S0165-0270(99)00129-6

Granger, C. W. (1969). Investigating causal relations by econometric models and cross-spectral methods. *Econometrica*, *37*, 424–438.

Lu, C., & Ferrier, N. J. (2004). Repetitive motion analysis: segmentation and event classification. In *IEEE Trans. Pattern Anal. Machine Intell.* (pp. 258–263).

Schwartz, G. (1978). Estimating the Dimension of a Model. *Ann. Stat.*, *5*, 461–464.
